# Quantum Chemistry-Based Prediction of Electron Ionization Mass Spectra for Environmental Chemicals

**DOI:** 10.1021/acs.analchem.4c02589

**Published:** 2024-08-07

**Authors:** Helge Hecht, Wudmir Y. Rojas, Zargham Ahmad, Aleš Křenek, Jana Klánová, Elliott J. Price

**Affiliations:** † RECETOX, Faculty of Science, Masaryk University, Kotlářská 2, Brno 602 00, Czech Republic; ‡ Institute of Computer Science, Masaryk University, Botanická 554/68a, Brno 602 00, Czech Republic

## Abstract

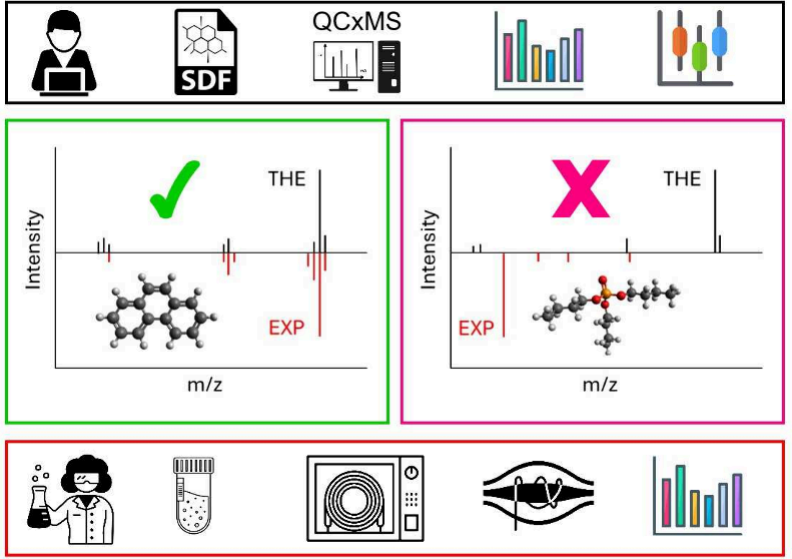

There is a lack of experimental electron ionization high-resolution mass spectra available to assist compound identification. The in silico generation of mass spectra by quantum chemistry can aid annotation workflows, in particular to support the identification of compounds that lack experimental reference spectra, such as environmental chemicals. We present an open-source, semiautomated workflow for the in silico prediction of electron ionization high-resolution mass spectra at 70 eV based on the QCxMS software. The workflow was applied to predict the spectra of 367 environmental chemicals, and the accuracy was evaluated by comparison to experimental reference spectra acquired. The molecular flexibility, number of rotatable bonds, and number of electronegative atoms of a compound were negatively correlated with prediction accuracy. Few analytes are predicted to sufficient accuracy for the direct application of predicted spectra in spectral matching workflows (overall average score 428). The *m*/*z* values of the top 5 most abundant ions of predicted spectra rarely match ions in experimental spectra, evidencing the disconnect between simulated fragmentation pathways and empirical reaction mechanisms.

## Introduction

Electron ionization (EI) mass spectra are widely used for the structural annotation of small molecules through comparison of experimentally acquired spectra with mass spectral libraries. EI is the most common ionization method coupled with gas chromatography– mass spectrometry (GC–MS). However, there is a lack of EI high-resolution mass spectral libraries available, especially for environmental chemicals.^1^ To date, the Thermo Contaminants Library v1.5 containing 951 analytes is the largest commercially available Orbitrap library of this kind. Community curated databases such as MassBank^2−5^ and GNPS^6^ currently do not contain any spectra acquired on GC-[Orbitrap] MS instruments. Additionally, spectra acquired using high-resolution time-of-flight (TOF) and Orbitrap instruments differ from those obtained through low-resolution single quadrupole instruments. The disparity leads to relatively lower match scores when comparing high-resolution spectra to nominal mass spectra, limiting the applicability of those libraries.^7,8^ Compounding this challenge, many chemicals are not readily available commercially, and existing experimental libraries exhibit limited chemical diversity.

As an alternative approach, spectra can be generated in silico and used for identification. Methods like machine learning (ML) or quantum chemistry (QC) calculations are employed for this purpose.^9^ For example, the neural electron–ionization mass spectrometry (NEIMS) software uses a multilayer perceptron (MLP) architecture to generate low-resolution EI mass spectra from molecular fingerprints.^10^ The specific model presented in the paper was trained in a supervised manner on the NIST/EPA/NIH Mass Spectral Library 2017 database. State of the art ML methods, such as graph neural networks (GNNs) and transformers, have demonstrated superior performance compared to MLP-based models^11^ and have been applied in various ways for mass spectra prediction tasks.^12−14^ In particular, Zhu and Jonas^15^ utilize a representation learning approach to predict the likelihood of subformulae within a predetermined depth of bond breakages. They then leverage these probabilities to scale the ion intensities in substructure spectra derived from isotopic patterns. Their study successfully predicted EI high-resolution mass spectra, including exact peaks, for molecules from PubChem, using an artificial high-resolution data set. However, despite these advancements, ML approaches typically require substantial amounts of high-quality training data. Consequently, the broader applicability of ML methods is hindered by the scarcity of available EI high-resolution mass spectral libraries, primarily limiting their usage to the prediction of low-resolution spectra.

In contrast, generating mass spectra through QC calculations does not rely on empirical rules or experimental data. Such approaches offer insights into fragmentation processes and reaction mechanisms. The quantum chemical electron ionization mass spectrometry (QCEIMS) software,^16^ later renamed QCxMS after the addition of collision-induced dissociation (CID) ionization and published as open-source,^17^ simulates the ionization and fragmentation process by employing QC principles to generate mass spectra in silico. Besides ab initio calculations,^16,18^ the package supports semiempirical quantum mechanical (SQM) methods^19,20^ for increased throughput. SQM modeling provides ionization potentials for high-temperature molecular dynamics (MD) and mass spectra modeling. The simplicity in parametrization, using hybrid density functional theory (DFT) reference data, enhances SQM’s accessibility and efficiency. Despite the potential overestimation of electron delocalization, SQM methods, such as GFN1-xTB, excel in handling metallic systems and covalent bond dissociation. However, GNF1-xTB requires adjustments to optimize directional electrostatic effects, such as halogen bonds.^21^ Conversely, GFN2-xTB does not necessitate these modifications for handling halogen bonds.^22^ A standout feature is SQM’s easily adaptable, element-specific parameters, enriching method versatility. Whereas DFT remains the most accurate, SQM-based MD is more than 100 times faster than pure DFT-based MD.

Several studies evaluate the performance of the QCxMS package and related SQM methods across multiple compound categories (Table 1). These include small data sets of (i) organic and inorganic small molecules used for method development validations,^23,24^ (ii) a restricted set of pollutants consisting of 27 halogenated compounds and 8 organophosphorus flame retardants (ODTs),^25^ and (iii) purines and pyrimidines.^26^ Notably, QCxMS has been recently applied to two larger data sets consisting of (iv) 451 small organic molecules^27^ and (v) 816 trimethylsilylated analytes.^28^

**Table 1:** Overview of Related Studies Using QCxMS with Semiempirical Calculations

study	classes	molecules	masses	method	reference	atom types
this study	56	367	108–715	GFN1-xTB	RECETOX Exposome HR-[EI+]-MS library^33^	C H O N F P S Cl Br Si
Wang et al.^27^	43	451	26–358	OM2	NIST17	C H N O
Wang et al.^24^	NA	41	55–333	OM2/CISD + GFN1-xTB	NIST17	C H N O F
Wang et al.^28^	10	816	115–299	GFN1-xTB	NIST17	C H N O Si
Schreckenbach et al.^25^	NA	35	74–959	GFN1-xTB	NIST14	C H N O P Cl Br
Ásgeirsson et al.^23^	NA	23	86–505	GFN1-xTB	NIST/SDBS^a^	C H N O P B Sb S Cl Bs Ge Te Ni Cu Cr Fe F Al Si Sn
Lee et al.^26^	12	80	120–207	OM2/OM3	NIST17	C H N O

a SDBS: https://sdbs.db.aist.go.jp/.

These studies demonstrate QCxMS’s ability to predict EI mass spectra, but the chemical space covered in the larger studies (containing ≥100 molecules) is limited to C, H, O, N, and Si atoms and molecular weight ≤400 Da. Notably, previous studies do not compare the predicted spectra against experimental high-resolution spectra acquired from analytical standards but against low-resolution commercial libraries, limiting the assessment of predictive accuracy.

Concerning the expansion of QCxMS applicability, it is crucial to highlight its limited use in investigating large diverse data sets including, for example, environmental chemicals^21^ (Table 2). In this Article, we introduce an open-source workflow for large-scale prediction of EI mass spectra, leveraging the QCxMS software and using the extended tight-binding GFN1-xTB^21^ method. The choice of GFN1-xTB aligns with methodologies used in prior studies on environmental chemicals.^25^ Although the OMx SQM methods perform better,^16^ they only support C, H, N, O, and F elements,^32^ limiting the applicable chemical space. We demonstrate the workflow through application to predict EI mass spectra for a previously published set of environmental compounds.^1^ Furthermore, we then investigate the applicability of this methodology on our chemically diverse data set by comparing the spectra to the accompanying high-resolution EI mass spectral library^33^ and outline aspects influencing prediction accuracy.

**Table 2:** Summary of Molecular Properties Investigated in Comparison to Previous Work^a^

		this study	Wang et al.^27^	Wang et al.^24^	Wang et al.^28^	Schreckenbach et al.^25^	Ásgeirsson et al.^23^	Lee et al.^26^
atoms	mean	33.01	21.95	22.82	**33.67**	31.03	19.76	18.28
	min	12	7	8	**17**	11	6	12
	max	**80**	59	56	58	74	49	24
aromatic nitrogens	mean	0.45	0.06	0	0.13	0.03	0	**1.39**
	min	**0**	**0**	**0**	**0**	**0**	**0**	**0**
	max	3	3	0	**4**	1	0	**4**
molecular complexity	mean	0.73	0.47	0.47	0.53	0.59	0.27	**0.76**
	min	0.38	0.12	0.27	0.26	0.35	0	**0.67**
	max	**1.18**	0.8	0.77	0.84	0.85	0.69	0.82
molecular flexibility	mean	0.36	0.33	0.3	**0.63**	0.51	0.4	0.04
	min	0	0	0	**0.22**	0	0	0
	max	0.85	0.86	0.69	**0.91**	0.9	0.89	0.23
rotatable bonds	mean	3.26	2.65	1.7	**4.41**	4.22	1.43	0
	min	0	0	0	**1**	0	0	0
	max	**21**	10	8	14	16	5	0
stereo centers	mean	**0.75**	0.66	0.38	0.33	0.59	0.29	0
	min	**0**	**0**	**0**	**0**	**0**	**0**	**0**
	max	**9**	6	3	4	8	6	0
electronegative atoms	mean	4.74	1.78	1.32	2.4	**5.94**	1.76	5.49
	min	0	0	0	1	2	0	**4**
	max	**14**	8	5	6	12	8	8

a Row-wise maxima are printed in bold. Molecular properties were computed using DataWarrior.^29^ Any molecules failing computation were excluded from the comparison. Structure databases in SDF format have been generated from the respective publications and are deposited on Zenodo.^30^ Additional chemical identifiers were collected using MSMetaEnhancer.^31^

## Methods

### Workflow

The developed semiautomated workflow for predicting mass spectra integrates various functions to streamline the process, significantly enhancing researchers’ capacity to conduct high-throughput mass spectra simulations. To the best of our knowledge, this addresses a deficiency in available open-source end-to-end processing solutions. It includes approaches such as managing input file preparation through templates and a global parameter file for every stage of the workflow on a high-performance computing (HPC) cluster. These are implemented as accompanying scripts in bash and python for batch job submission, progress monitoring (i.e., to obtain statistics on job success or failure), error handling (e.g., scripts to check GAMESS conformer generation or to resubmit jobs), result collection, and data analysis. This ensures comprehensive monitoring and reliability of the computational process.

**Figure 1:**
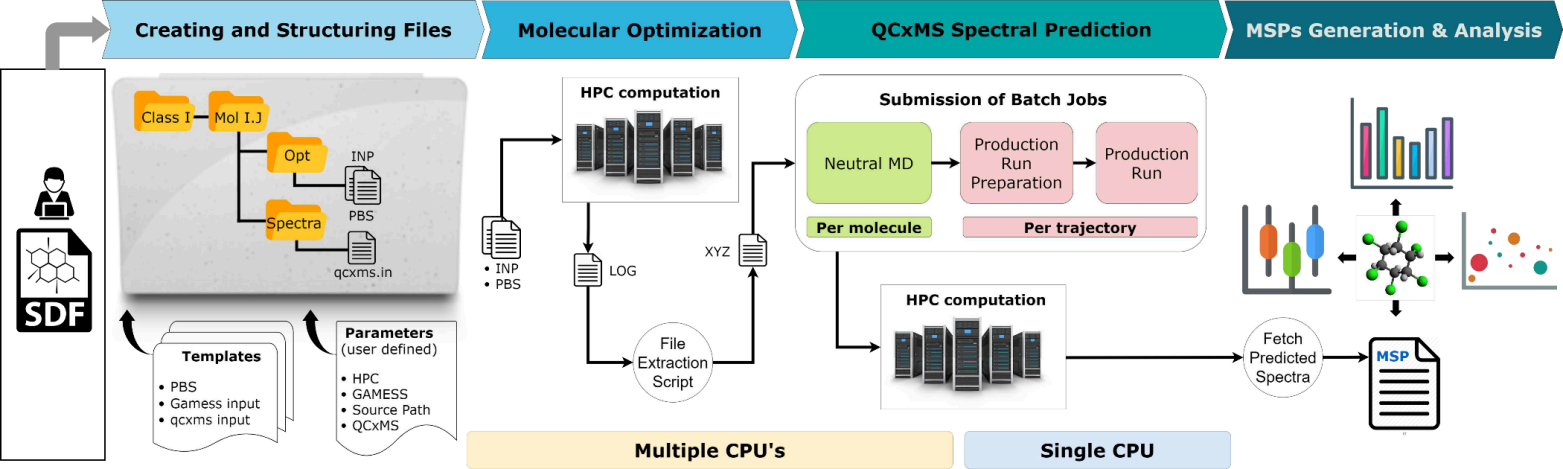
Semiautomatic EI spectra prediction workflow. From an SDF input, the workflow creates all input files required for batch spectral prediction. The workflow is open-source and publicly available online.^30^

Starting with the structure data file (SDF) input, the workflow progresses through these steps: (i) Creating and Structuring Files: arranging files for molecular geometry optimization with GAMESS,^34^ handling molecule reading, directory creation, 3D conformation generation, input file writing, and optimization job script setup; (ii) Molecular Optimization: dispatches optimization tasks to the HPC cluster and gathers optimized molecular inputs for spectral simulations; (iii) QCxMS Spectral Prediction: submits neutral MD, prepares production runs, and executes them on the HPC cluster using batch job processing; and (iv) MSPs Generation and Analysis: retrieves spectral results and formats them into an .msp file. Additionally, the workflow includes postprocessing tools for summarizing simulations and removing unnecessary files. It offers visualization and analysis tools for enhanced usability. A high-level overview of this workflow is illustrated in Figure 1.

### Data Set

The RECETOX Exposome HR-[EI+]-MS library^33^ was used as the data source. The library contains 56 distinct chemical classes, covering molecules ranging from 12 to 80 atoms, representing a diverse range of compounds in terms of both size and elemental composition^1^ (Table 2). Isotopically labeled analytes were removed from the library as those are not handled by QCxMS, leaving 367 structures.

### 3D Conformer Generation

Molecular structures and other metadata, for example, ChemOnt chemical class,^35^ were extracted from the RECETOX Exposome HR-[EI+]-MS library^33^ in SDF using the RDKIT package.^36^ Subsequently, three-dimensional conformers were created from simplified molecular input line entry system (SMILES) descriptors using a customized variant of the rdconf code (see https://github.com/dkoes/rdkit-scripts/blob/master/rdconf.py), which leverages RDKIT functions while employing the universal force field (UFF) method. Molecular structures were optimized using GAMESS via a self-consistent field (SCF) wave function calculation employing the Restricted Hartree–Fock (RHF) method at the 6-31G level of theory, with the optimization employing a gradient convergence criterion of 5 × 10^–4^ prior to QCxMS analysis. While this workflow for conformer generation and geometry optimization can be applied to molecules with a broad range of elements in the periodic table (up to *Z* = 86), this may not hold true for molecules with complex electronic structures.

### Spectra Prediction Using QCxMS

QCxMS calculations were performed using default parameters (electron energy, 70 eV; excess energy, 0.6 eV/atom; initial temperature, 500 K; simulation time, 10 ps). The ground-state trajectories and production runs were performed using the SQM GFN1-xTB method. After fragmentation, the ionization energies (IEs) were calculated using GFN1-xTB, and the fragment with the lowest IE obtained the positive charge. A default number of trajectories were computed (25× the number of atoms in the molecule) for every molecule.

### Postprocessing

Results were collected and converted into a library in the .msp format, and the predicted spectra underwent further filtering using the matchms^37^ package. Filtering steps were tailored to ensure reliable comparison between predicted spectra and reference spectra: (i) the *m*/*z* values were restricted to the range of 70–700, (ii) peak intensities were normalized to the peak of maximum intensity, and (iii) peaks with intensities below 1% of the maximum peak intensity were removed. A second data set containing the top 5 intensity ions from both predicted and experimental spectra was created because, in practice, only minimal spectral information is often available for annotation of compounds in experimental data sets. Chemical properties related to atomic composition and molecular structure were computed using DataWarrior^29^ (see Table 2). Statistical analysis was performed using scipy^38^ and pandas.^39^

### Spectral Matching

Spectral matching was performed using matchms. The *CosineHungarian* score was used with a tolerance of 0.0035 Da (i.e., 5 ppm at 700 *m*/*z*), intensity power of 1, and *m*/*z* power of 0. In spectral similarity computation, only peaks that fall within the specified tolerance are taken into account. After this filtering process, spectrum vectorization can be understood as the intersection between the compared spectra. Therefore, peaks not present in both spectra do not negatively impact the scoring. This is different from other commonly used scoring methods (e.g., forward and reverse cosine similarity,^40^ hybrid search^41^ as employed by NIST MSSearch or spectral entropy similarity^42^), which might be affected by the presence or absence of peaks. Spectral entropy scores calculated were found to correlate with the matchms cosine score-based matching (Pearson correlation of 0.87).

As matchms does not retain entries with 0 matching ions in the outputs, these were systematically integrated into the score tables, assigning scores and match values of zero for pairs missing in the output files. The workflow for spectral matching and related data sets are available online (i.e., top 5 intensity^43^ and all peaks^44^ spectral similarities). For more details regarding the different scoring methods, we refer the reader to the online resources.^30^

## Results

All computations were conducted on the metacentrum HPC cluster (MetaCloud; https://www.metacentrum.cz/en/cloud/).

### Geometry Optimization

A subset of 48 molecules did not converge using the previously described geometry optimization parameters (see failed_optimized_mols.csv^30^). This is most likely due to the generated conformer resulting in an unfavorable initial configuration for geometry optimization. The computational framework allows users to adjust key parameters such as basis sets, gradient convergence tolerance, and the maximum number of steps to address convergence challenges. However, our primary focus at this stage is to enable the generation of molecular structures for spectral predictions, rather than achieving high-level molecular optimization. Therefore, users also have the flexibility to employ alternative tools for refining these molecular structures. In this context, we leveraged the xTB code^45^ to address the molecular optimization of the aforementioned molecules.

### Accuracy of Predicted Spectra

**Figure 2:**
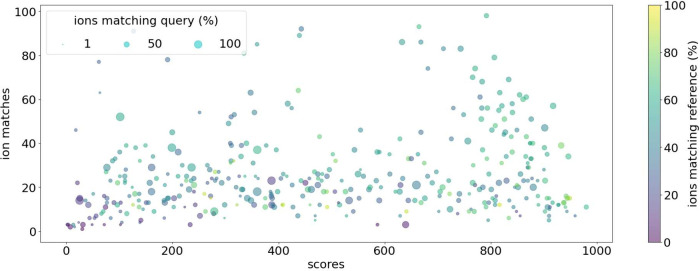
Cosine similarity scores (*x*-axis) versus the absolute number of matched ions (*y*-axis) for predicted and experimental spectra. The color scale and size represent ion matches in % normalized by the number of peaks contained in the experimental (query, size) and predicted spectrum (reference, color), respectively.

A comparison between the predicted and experimentally acquired spectra, based on intensity ratio similarity (cosine score) and the number of matching ions, does not show any direct trend indicating the failure of the method to achieve meaningful predictions, with the scores and ion matches heterogeneously distributed (Figure 2).

### Physicochemical Properties

**Figure 3:**
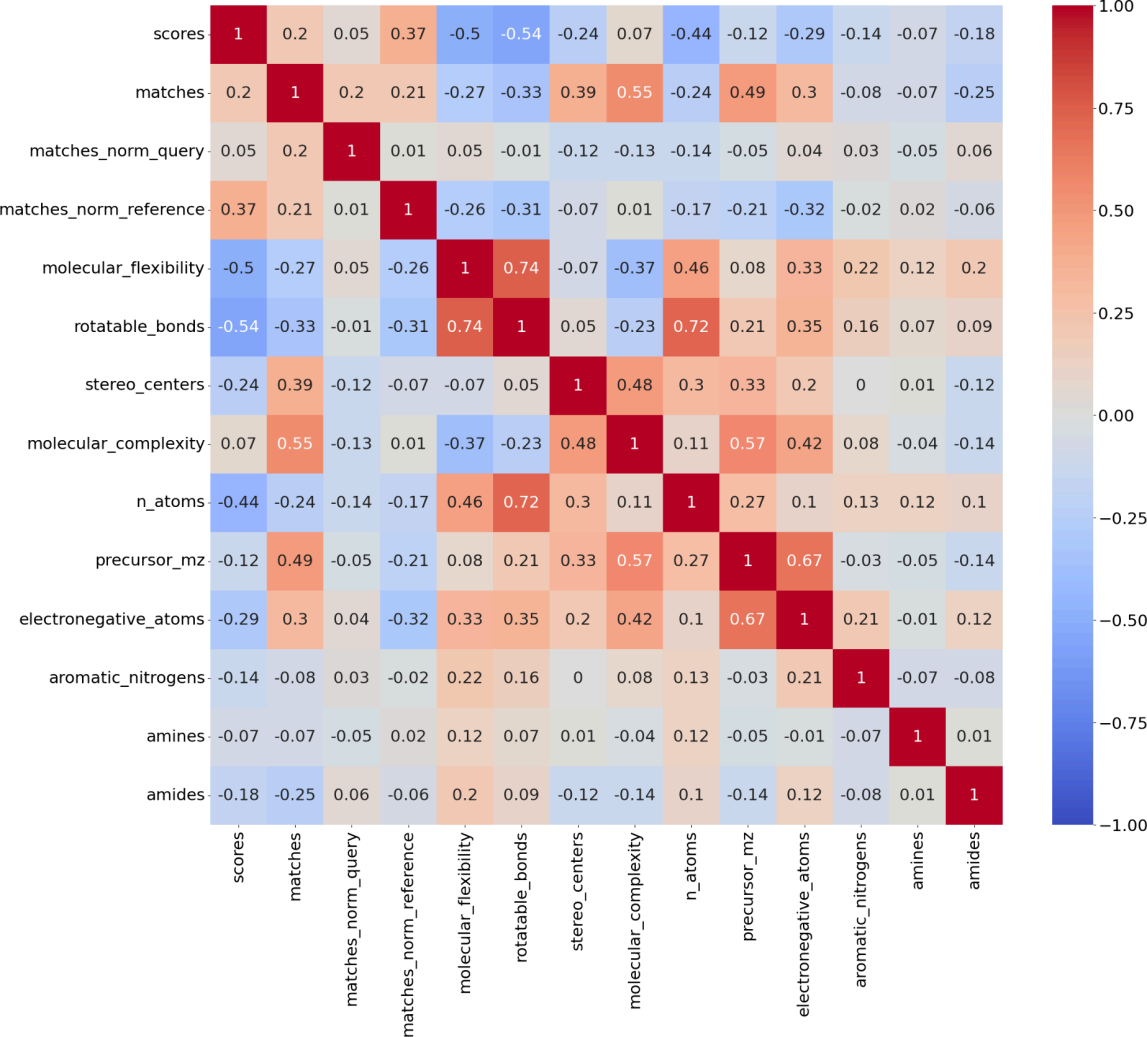
Pearson’s correlations of chemical properties and spectral matching results. Chemical properties were computed using DataWarrior. Molecular complexity is used as an aggregate measure.^46^ The number of ion matches is given as absolute number as well as normalized by the number of peaks in the predicted and reference spectra.

Investigating the correlations of spectral matching scores and computed chemical properties reveals a weak positive correlation between cosine similarity score and number of ion matches. Furthermore, there is a negative correlation between scores and molecular flexibility, the number of rotatable bonds, the number of atoms, and the number of electronegative atoms. These properties are cocorrelated. The number of matching ions follows a similar trend. However, it shows weaker correlation with the number of atoms. Notably, molecular complexity does not correlate with the cosine similarity score, nor the number of matched ions (Figure 3).

### Chemical Class

**Figure 4:**
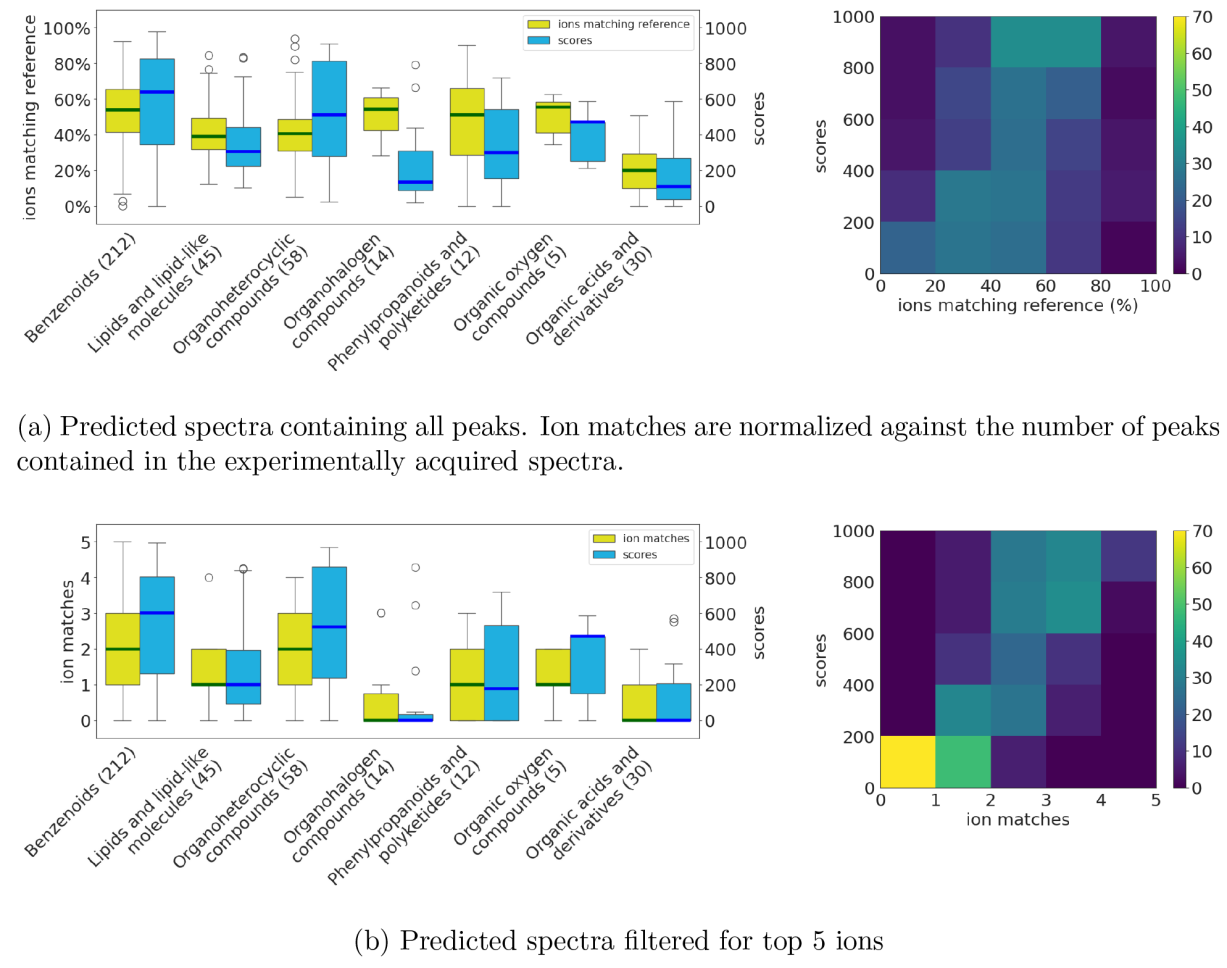
Spectral matching of predicted to experimental spectra at the superclass level. The color scale for counts has been aligned across histograms.

Comparisons were performed based on ChemOnt^35^ chemical class hierarchies to investigate prediction accuracy among structurally related groups (Figure 4).

At the superclass level, only the benzenoids and organoheterocyclic compounds have a median spectral cosine score greater than 500 and an interquartile range (IQR) exceeding 800. However, the prediction accuracy for lipid and lipid-like molecules, organohalogen compounds, phenylpropanols, and polyketides, as well as for organic acids and derivatives, is comparatively lower, with all having median scores below 400. The lipid and lipid-like molecules are characterized by a high number of rotatable bonds (on average ≥7) and contain additional atoms beyond C, O, and H such as P and S. The organic acids superclass shows the lowest median cosine similarity score of 110.5, encompassing most S- and P-containing molecules in the data set. Low spectral similarity scores have previously been demonstrated for ODTs.^25^ Particularly for those structures containing S and P, the SQM methods show poor accuracy in simulating fragmentation pathways.

Overall, when considering only the top 5 intensity ions, the spectral match between predicted and experimental spectra is even lower (Figure 4b). For 71 spectra, not a single of the top 5 experimentally measured ions is predicted at the correct *m*/*z*. In practice, three or more ions are often used to corroborate annotation, yet three or more ions are only predicted correctly for 95 of the 367 molecules.

**Figure 5:**
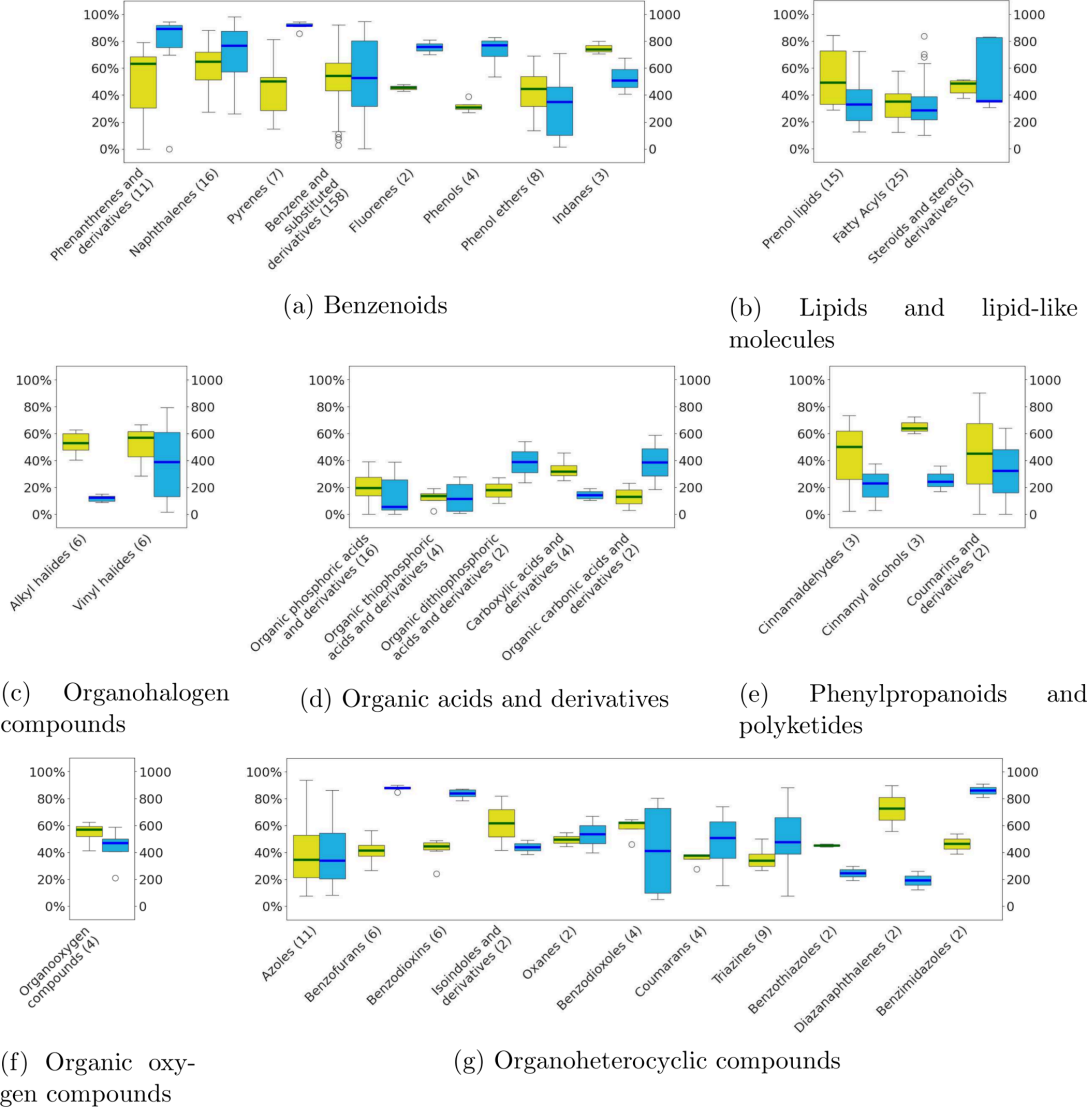
Boxplot of spectral comparisons between predicted and reference EI mass spectra per chemical class within each superclass. The bar denotes the median, while outliers are depicted as circles with spectral similarity score (blue, right *y*-axis) and percentage of ions matching the reference spectra (yellow, left *y*-axis) displayed. Superclasses and classes (*x*-axis) represented only by a single molecule are excluded. Tables summarizing the mean and median scores, matches, and physicochemical properties are available on Zenodo.^30^

On the class level (see Figure 5), two groups stand out for their high prediction accuracy: (i) phenanthrenes and pyrenes (benzenoids), and (ii) benzofurans, benzodioxins, and benzimidazoles (organoheterocyclic compounds), totaling 34 molecules. These molecules, characterized by their relatively planar geometry (molecular flexibility ≤0.4), were reliably predicted with median scores ≥800. However, certain individual classes showed lower prediction performance, indicated by median scores ≤350. These include (i) phenol ethers (benzenoids), (ii) alkyl halides (organohalogen compounds), and (iii) azoles, benzothiazoles, and dizianaphthalenes (organoheterocyclic compounds).

In comparison to other benzenoids, the phenol ethers in the data set consistently exhibit at least three rotatable bonds, with some also containing aromatic nitrogens or being halogenated. Similarly, the organoheterocyclic classes possess a higher count of aromatic nitrogens and rotatable bonds (both ≥2), along with electronegative atoms (≥5). Additionally, benzothiazoles, which contain S, are not effectively characterized by the SQM methods.

**Figure 6:**
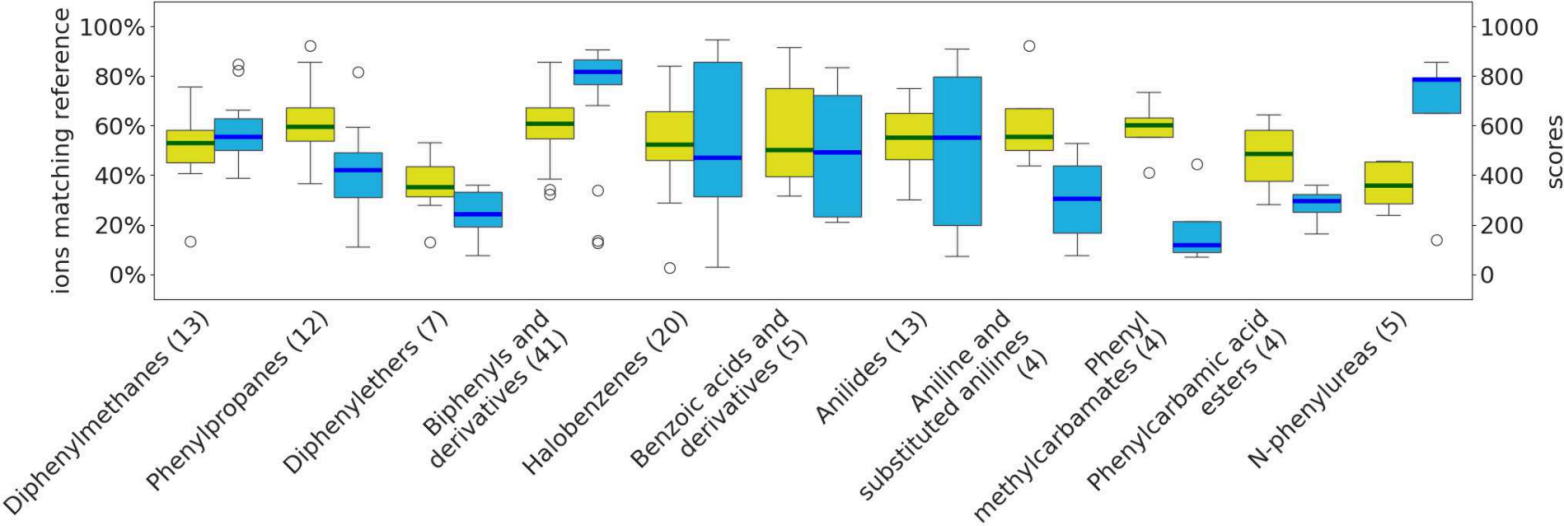
Boxplots for subclasses of benzene and substituted derivatives. The bar denotes the median, while outliers are depicted as circles. Subclasses with less than three molecules have been removed from the figure.

As benzenes and substituted derivatives consist of 158 molecules, we further inspected those at the subclass level (Figure 6). Biphenyls and derivatives constitute the largest subclass, with 41 molecules, and are most accurately predicted, with a median cosine similarity above 800. The outliers such as bitertanol (3,3-dimethyl-1-(4-phenylphenoxy)-1-(1,2,4-triazol-1-yl)butan-2-ol) and bifenazate (propan-2-yl *N*-(2-methoxy-5-phenylanilino)carbamate), each having six rotatable bonds, are observed within this subclass. Accordingly, subclasses containing molecules with greater structural flexibility, such as phenyl methylcarbamates, phenyl methylcarbamic acids, and diphenylethers, are predicted less accurately.

### Elemental Composition

The class-based analysis reveals a trend that spectra prediction of molecules containing certain atom types, especially aromatic nitrogens present in compounds like azoles, as well as the presence of P, is less accurate. Therefore, in addition to the class-based analysis, we also analyzed the spectra matching results regarding the elemental composition of each molecule.

**Figure 7:**
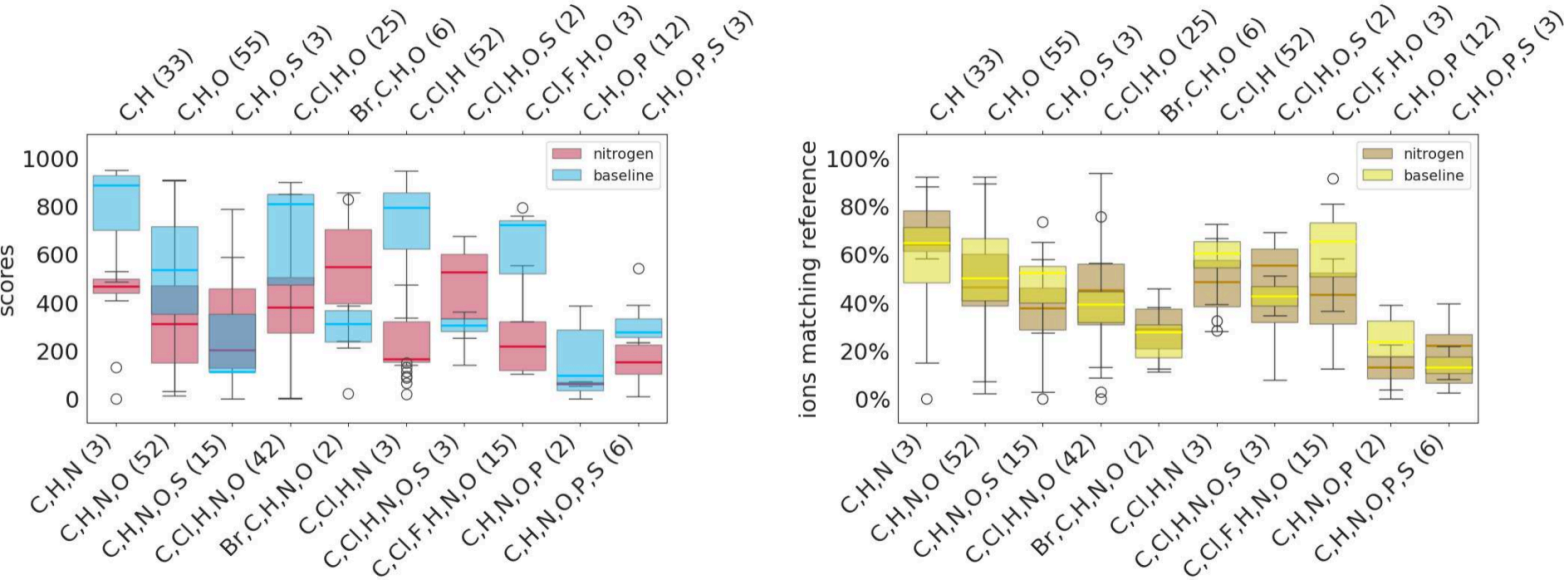
Comparison of spectra prediction accuracy for molecules of selected chemical compositions based on the presence of N. The selected groups of molecules contain all of the form X → X + N present in the data set.

To isolate the influence resulting from the presence of N in the molecule, we directly compared molecules containing the same atom types but with and without N (Figure 7). Median scores and ion match rates are lower for every group of molecules, except for those containing the chemical compositions (i) Br,C,H,(N),O; (ii) C,H,(N),O,S; and (iii) C,Cl,H,(N),O,S, when N is part of the chemical composition.

**Figure 8:**
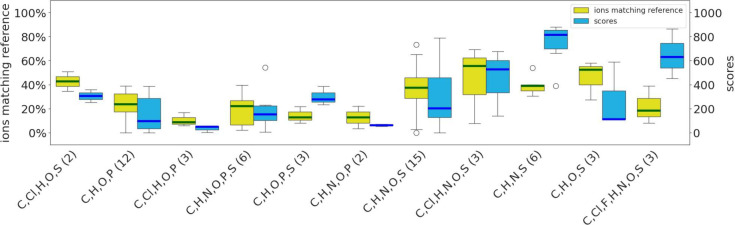
Results for molecule groups containing P and/or S atoms. Groups consisting only of a single molecule have been removed from the boxplot.

Similarly, the presence of P negatively influences prediction accuracy using our chosen methodology (Figure 8). P often serves as a central atom, resulting in a more flexible structure. Within our data set, P containing molecules have an average number of rotatable bonds of 8.4 over 2.9 for all other molecules, respectively. This is further supported by the negative correlation of the rotatable bond count and cosine similarity and number of matching ions (Figure 3).

## Discussion

Our results show that performance of QCxMS using the GFN1-xTB methods for the prediction of EI mass spectra varies significantly across the diverse data set. Only few chemical groups (e.g., pyrenes, phenanthrenes, benzofuranes, benzodioxins, benzimidazoles, and biphenyls and derivatives, totaling 75 molecules) are predicted with sufficient accuracy (i.e., average spectral similarity scores above 800) for direct use in spectral matching-based annotation workflows. In particular, for the vast majority of analytes, even the most abundant ions were not accurately predicted (i.e., wrong *m*/*z*), limiting the current potential to incorporate predicted spectra into suspect annotation workflows.

The presence of atoms other than C, O, and H leads to less accurate spectral prediction, as can be seen on the results of P- and/or S-containing molecules depicted in Figure 8 and the comparison of N-containing groups and their respective N-free counterparts in Figure 7. Also, Wang et al.^27^ report comparably lower scores even for O-containing molecules. Additionally, the extensive presence of electronegative atoms such as halogens (e.g., in alkyl and vinyl halides, see Figure 5c) negatively affects the simulations, as halogen bonds typically require atom pairwise corrections.^21^ We repeated predictions of the alkyl halides using the GFN2-xTB method that models anisotropic electrostatic interactions and does not employ specific element pair corrections. Although it reportedly improves predictions of molecules containing bonds between molecules with large differences in electronegativity,^22^ we did not observe any improvement in spectra quality for our subset.^48^ Beyond chemical composition, the related three-dimensional molecular structure is the main determinant. While planar geometries are predominantly predicted with high accuracy, central atoms with many rotatable bonds (e.g., P in organic acids, see Figure 5d) and other stereo centers lead to lower prediction accuracy for the QCxMS method.

**Figure 9:**
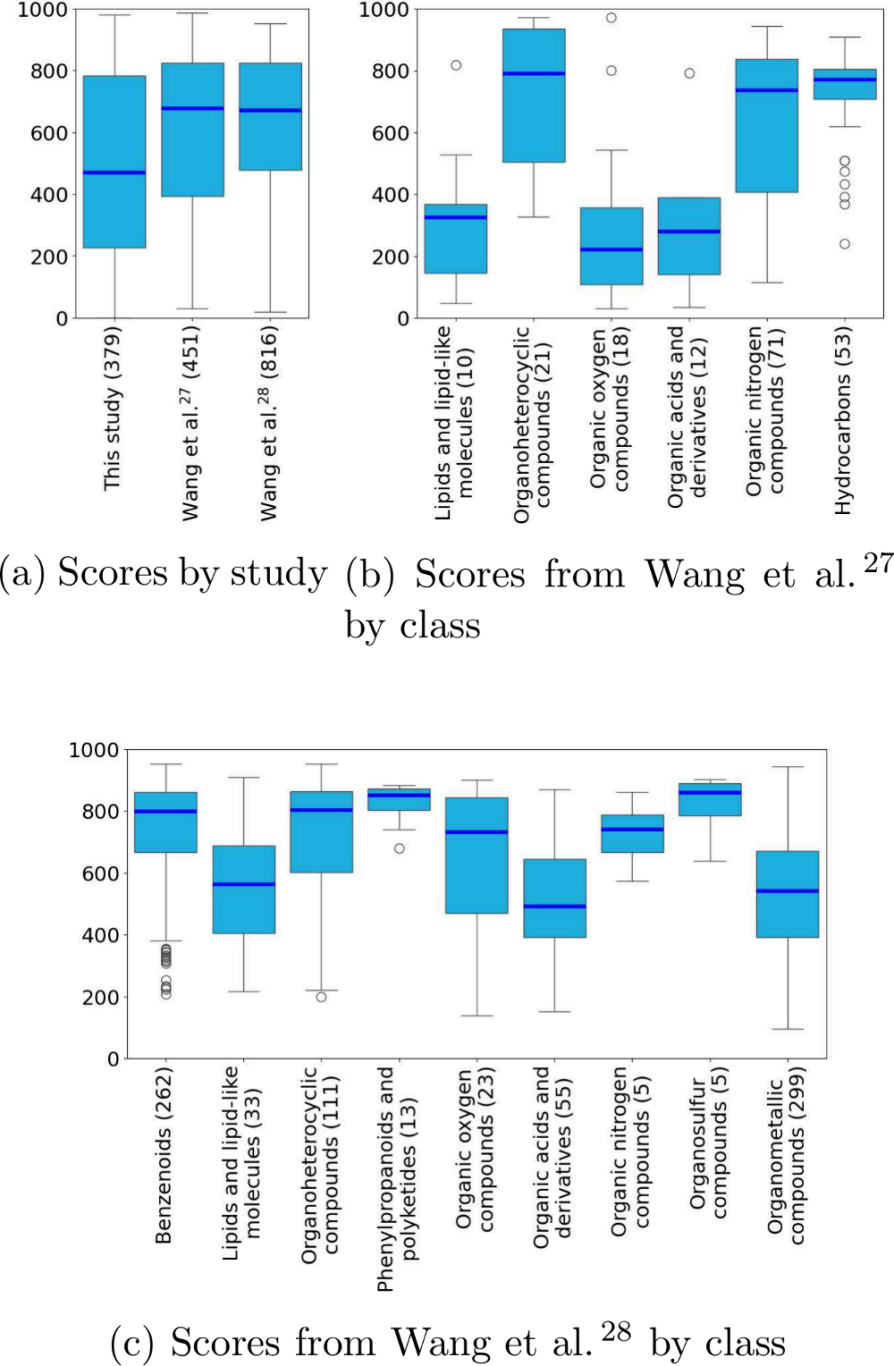
Distributions of scores across studies (a) and from other large studies^27,28^ based on the same ChemOnt classification (b,c). SMILES identifiers were collected using MSMetaEnhancer, and molecules were classified using ClassyFire. Tables used for the plots are available under analysis/data/reference online.^30^

These findings are in line with previous studies reporting very low matching scores for ODTs^25^ and lower scores obtained for aliphatic molecules over aromatic ones.^28^ In contrast to Wang et al.,^27^ we found molecular flexibility to negatively correlate with prediction accuracy. Although a similar trend (Pearson correlation of −0.3 between the dot product score and the number of rotational bonds) appears in their data as well (see comparisons.ipynb^30^), this might be related to the differences between the GFN1-xTB and OM2 SQM methods. Previous research relied on differing metrics to evaluate spectra prediction quality, typically comparing data solely against low-resolution mass spectra.^23−28^ To put the results from the previous larger studies into context, we conducted the same classification-based analysis (Figure 9).

The observed limitations with regard to the chemical space for which accurate predictions are possible hinder application for many parent environmental chemicals, which are enriched with halogens and/or S compared to endogenous analytes. In addition, the spectral prediction for many metabolites is likely to also be poor due to their higher proportion of P and rotatable bonds.^49^ However, previous investigations to predict spectra of endogenous analytes rarely included P (Table 1). Simply tuning simulation parameters may not significantly enhance predictions; instead, it is proposed that accurate calculation of potential energy surfaces or the incorporation of excited-state MD may enhance accuracy within the semiempirical framework.^50^ Recently, Wang et al.^24^ incorporated excited states into the MD steps of EI spectra using the binary-encounter-Bethe (BEB) model, achieving higher accuracy, although limitations still exist, particularly for nonorganic molecules.

The semiempirical xTB method enables systematic processing of larger sets of molecules, although true high throughput processing would require a speedup in several orders of magnitude. Including initial testing, we scheduled 524 334 compute jobs with a total usage of 43 201 central processing unit (CPU) days on our HPC cluster. The files containing logs, structures, and computed trajectories require ∼2 TB storage space. Considering that such infrastructure and capacity might not be generally available to researchers further highlights the need for more flexible, accessible, and efficient computational methods.^51,52^ For similar reasons, current works are limited to the use of SQM over DFT-based methods. Future work should consider the application of such ab initio MD for molecules not well characterized by the SQM-based methods.

## Conclusions

We present an open-source workflow for the prediction of high-resolution EI mass spectra and performance assessment based on the QCxMS, matchms, RDKIT, xTB, and GAMESS software packages. Additionally, we provide the largest set of predicted mass spectra of environmental chemicals so far and perform an unbiased analysis based on structural taxonomy classifications and chemical element composition. Our results show that further developments of SQM-based MD to improve prediction accuracy for molecules containing electronegative atoms (e.g., halogens, nitrogen) with high molecular flexibility (e.g., multiple rotatable bonds and stereo centers) are crucial. Current methods are insufficient for practical suspect screening applications, which require at least three characteristic (i.e., highly abundant) ions for correct identification, as these conditions were not met for the vast majority of predicted spectra when compared with experimental high-resolution spectra. Furthermore, improvements with regard to computational efficiency and accessibility are essential to advance the field of QC-based in silico mass spectra prediction. This especially holds true considering the need for additional comprehensive large-scale studies, which are required to characterize the applicable chemical space and potential pitfalls of these methodologies.

## Data Availability

All data and scripts used in this work are hosted and archived on Zenodo.^30,33^ Mirrorplots for all corresponding pairs of predicted and experimental spectra are also available on Zenodo.^53^ Spectral similarity computations and related results that were created using Galaxy are archived as Galaxy histories, which can be imported into any Galaxy instance.^43,44^ The code repository containing the workflow as well as the scripts used to generate tables and figures is publicly available at https://github.com/RECETOX/ei_spectra_predictions. Note that this repository is subject to further development, so please refer to the Zenodo archive for information specifically related to this publication.

## Glossary

BEB: binary-encounter-Bethe
CID: collision-induced dissociation
CPU: central processing unit
Da: dalton
DFT: density functional theory
EI: electron ionization
GC–MS: gas chromatography–mass spectrometry
GNN: graph neural network
HPC: high-performance computing
IE: ionization energy
IQR: interquartile range
MD: molecular dynamics
ML: machine learning
MLP: multilayer perceptron
NEIMS: neural electron–ionization mass spectrometry
ODT: organophosphorus flame retardant
QC: quantum chemistry
QCEIMS: quantum chemical electron ionization mass spectrometry
RHF: restricted Hartree–Fock
SCF: self-consistent field
SDF: structure data file
SMILES: simplified molecular input line entry system
SQM: semiempirical quantum mechanical
TOF: time-of-flight
UFF: universal force field
